# Detection of infectious SARS-CoV-2 in ocular samples is linked to viral load in the nasopharynx

**DOI:** 10.3389/fcimb.2024.1332157

**Published:** 2024-03-04

**Authors:** Janine Kimpel, Annika Rössler, David Bante, Wegene Borena, Dorothee von Laer, Claus Zehetner, Teresa Rauchegger, Stefanie Seiwald, Barbara Falkensammer

**Affiliations:** ^1^ Institute of Virology, Medical University of Innsbruck, Innsbruck, Austria; ^2^ Center for Virology and Vaccine Research, Beth Israel Deaconess Medical Center, Harvard Medical School, Boston, MA, United States; ^3^ Department of Ophthalmology, Medical University of Innsbruck, Innsbruck, Austria; ^4^ Department of Internal Medicine III, Medical University of Innsbruck, Innsbruck, Austria

**Keywords:** SARS-CoV-2, COVID-19, nasopharyngeal swab, ocular swab, virus culture, Oxford Nanopore Technology, whole genome sequencing, ocular transmission

## Abstract

**Introduction:**

SARS-CoV-2 is known to infect respiratory tissue cells. However, less is known about infection of ocular tissue and potential infectivity of lacrimal fluid. With this study, we want to compare viral loads in eye and nasopharyngeal swabs and analyze these for infectious virus.

**Methods:**

Between May 2020 and April 2021 ocular and nasopharyngeal swabs were collected from 28 SARS-CoV-2 infected patients treated on the corona virus disease 2019 (COVID-19)-ward of the University Hospital of Innsbruck, Austria. Samples with PCR detectable SARS-CoV-2 were analyzed via whole genome sequencing and an attempt was made to isolate infectious virus.

**Results:**

At the time point of sample collection, 22 individuals were still PCR positive in nasopharyngeal samples and in 6 of these patients one or both ocular samples were additionally positive. CT-values in eyes were generally higher compared to corresponding nasopharyngeal samples and we observed a tendency for lower CT-values, i.e. increased viral load, in nasopharyngeal swabs of individuals with at least one infected eye, compared to those where ocular samples were PCR negative. Ocular and nasopharyngeal sequences from the same patient were assigned to the same variant, either the D614G or the Alpha variant. Infectious virus was successfully isolated from 9 nasopharyngeal swabs, however only from one of the seven PCR positive ocular samples.

**Conclusion:**

We could detect SARS-CoV-2 in eyes of some of the infected patients albeit at lower levels compared to nasopharyngeal swabs. However, our results also indicate that lacrimal fluid might be infectious in patients with high viral load.

## 1 Introduction

Before December 2019, six human-pathogenic coronaviruses were described. Four of them, NL63, 229E, HKU1 and OC43, are known as common cold viruses and usually only cause mild symptoms (van der Hoek et al., 2004; Lau et al., 2006). Two additional viruses, the severe acute respiratory syndrome corona virus-1 (SARS-CoV-1) and the Middle East respiratory syndrome corona virus (MERS-CoV) were described in 2003 (Zhong et al., 2003) and 2012 (Zaki et al., 2012) respectively and can cause a wider range of disease severity ranging from asymptomatic to severe disease and death.

Since December 2019 another coronavirus spread rapidly across the globe causing mild to severe and deep respiratory infections (Zhou P. et al., 2020). On January 30^th^ 2020 the World Health Organisation (WHO) officially declared the coronavirus disease 2019 (COVID-19) pandemic as an international concern caused by the severe acute respiratory syndrome coronavirus 2 (SARS-CoV-2) (Eurosurveillance Editorial Team, 2020). SARS-CoV-2 infects lower and upper respiratory epithelial cells and uses angiotensin-converting enzyme 2 (ACE2) as primary receptor (Lan et al., 2020). Not only cells in the respiratory track but also the eye can express ACE2 and might consequently be infected by SARS-CoV-2 (Senanayake et al., 2007). In line, SARS-CoV-1 and SARS-CoV-2 were detected in previous studies in rare events via PCR in ocular samples of infected patients (Leong et al., 2004; Loon et al., 2004; Al-Sharif et al., 2021). Conjunctivitis and other ocular pathologies have also been reported as symptoms associated with SARS-CoV-2 infection (Cavalleri et al., 2020; Wu et al., 2020; Harthaller et al., 2022). However, still little is known about ocular infection of SARS-CoV-2. We therefore aimed here to analyze viral load and infectivity of matching samples from nasopharyngeal and ocular swabs.

## 2 Materials and methods

### 2.1 Patients

The ethical committee (EC) of the Medical University of Innsbruck, Austria, had approved the study protocol (EC number 1104/2020). Written informed consent was obtained from all study participants. Between May 2020 and April 2021 ocular and pharyngeal swabs were taken from 28 patients infected with SARS-CoV-2 and hospitalized at the COVID-19 unit of the University Hospital of Innsbruck, Austria. From each patient three swabs were taken from the right and left conjunctiva as well as from the pharynx.

### 2.2 Samples and viral RNA detection

Swabs were stored in a viral transport medium (containing Hanks Balanced Salt Solution (1x), 2% sterile, heat-inactivated Fetal Bovine Serum, 100 µg/mL Gentamicinsulfate, 0.5 µg/mL Amphotericin B) and sent to the Institute of Virology of the Medical University of Innsbruck, Austria. Samples were processed in the laboratory within two to six hours. Total nucleic acid extraction from swab samples was done using the NucliSENSE Kit with the EasyMag platform (bioMérieux, Marcy l’Etoile, France). Remaining swab samples were stored at -80°C for later virus culture attempts. Samples obtained from viral culture attempts were extracted by mixing the culture supernatant in a 1:1 ratio with DLR buffer (0.5% IGEPAL, 25 mM NaCl in 10 mM Tris-HCl buffer, 15 µL RiboLock RNase Inhibitor (ThermoScientific, 40 U/µL, EO0381) per mL DLR). Subsequently, PCR was performed with the RealStar SARS-CoV-2 RT-PCR kit 1.0 (Altona Diagnostics, GmbH, Hamburg, Germany) using a BioRad CFX96TM Real-Time System (Bio-Rad, Feldkirchen, Germany) according to the manufacturers’ instructions. CT (cycle threshold)-values below 40 were rated as positive.

### 2.3 Cell line

Vero cells stably overexpressing TMPRSS2 and ACE2 receptor (referred to as Vero-TMPRSS2/ACE2) and therefore highly susceptible to SARS-CoV-2 (Riepler et al., 2020), were maintained in DMEM (Dulbecco`s Modified Eagle Medium) supplemented with 10% FCS (fetal calf serum), 2% L-Glutamine (200 mM), 1% Penicillin-Streptomycin (10,000 U/mL), 1% MEM non-essential amino acids solution (100x), 1% Sodium Pyruvate, and selected with 10 µg/mL blasticidin and 500 µg/mL hygromycin. For SARS-CoV-2 infection experiments, FCS concentration was reduced to 2% and antibiotics were omitted. Cells were incubated in humidified incubators at 37°C and 5% CO_2_.

### 2.4 Viral culture

To evaluate the presence of infectious SARS-CoV-2, we performed a virus isolation protocol. Therefore, we seeded Vero-TMPRSS2/ACE2 cells in 6-well plates (3 x 10^5^ cells in 2 mL per well) the day before isolation attempts. Thawed swab samples were mixed with medium and filtered through 0.45 µm pore Costar Spin-X^®^ centrifuge tubes for 5 min at 4,000 rpm in a tabletop centrifuge. The filtrate was filled to a total volume of 1 mL with medium, before the supernatant of sub-confluent Vero-TMPRSS2/ACE2 cells was aspirated and replaced by the filtrate/medium mix. Cells were inoculated for 2 h at 37°C and 5% CO_2_. Subsequently, the filtrate/medium mix was replaced by fresh medium and cells were incubated for three days before the cytopathic effect (CPE) of cells was evaluated microscopically. Virus isolations were rated positive when a clear CPE was visible, while absence of CPE was considered negative. We previously showed that the CPE was much more pronounced on Vero-TMPRSS2/ACE2 cells than on parental Vero cells making it easier to recognize productively infected wells (Riepler et al., 2020). Supernatants of CPE positive wells were collected, centrifuged and stored at -80°C, whereas supernatants of wells without clear CPE were transferred to freshly seeded cells for a second isolation passage. After three days, cells were analyzed for CPE and supernatants were frozen down as described before. To validate successful virus isolation, a SARS-CoV-2 specific PCR was performed. Additionally, a TCID_50_ assay was performed to confirm the presence of infectious virus as previously described (Riepler et al., 2020).

### 2.5 Nanopore sequencing

For sequencing, total nucleic acid was extracted from samples or cultured virus using the EasyMag platform as described above. Sequencing libraries were prepared according to the Midnight Protocol by ONT (Oxford Nanopore Technologies, Oxford, UK), which is adapted from Freed et al (Freed et al., 2020), using the Midnight-IDT/V1 primer set from IDT (Integrated DNA Technologies, Coralville, Iowa, United States), or the VarSkipShort (v1a) primers from NEB (New England Biolabs, Ipswich, Massachusetts, United States) for better coverage, and the Rapid Barcoding Kit SQK-RBK110.96 for sequencing with R9.4.1 flow cells on the MinION Mk1B platform (ONT). Sequence analysis was performed using the epi2me-labs/wf-artic (ARTIC SARS-CoV-2 workflow and reporting) nextflow workflow (Ewels et al., 2020), which is based on the ARTIC Network bioinformatics pipeline for SARS-CoV-2 (Artic Network).

## 3 Results

Nasopharyngeal as well as conjunctival swab samples from both eyes were collected from 28 hospitalized COVID-19 patients. Patients had a mean age of 66.7 years and around half of them were male. Patient characteristics are specified in **Table 1**. Disease progression of every single participant was assigned according to the COVID-19 severity score (WHO Working Group on the Clinical Characterisation and Management of COVID-19 infection, 2020), which ranges from 0 (not infected) to 10 (dead). In this study group 82.1% suffered from COVID-19 pneumonia and the majority of patients (95.8%) required oxygen. 26 patients recovered from COVID-19 and could be dismissed from hospital after a mean duration of 13.4 days.

**Table 1:** Patients characteristics.

Characteristic	Study population N = 28^#^
Mean age ± SD (Range) [years]	66.7 ± 13.8 (38-92)
Number of males (%)	13 (46.4)
Underlying conditions	
Respiratory disease N (%)	7 (25.0)
Cardiovascular disease N (%)	18 (64.3)
Diabetes mellitus N (%)	7 (25.0)
Obesity N (%)	3 (10.7)
Oncologic disease N (%)	6 (21.4)
Mean duration stay on the COVID-19 ward ± SD (Range) [days]	13.4 ± 5.2 (5-26)
Mean COVID-19-Severity Score (WHO) ± SD (Range)^§^	5.3 ± 1.8 (1-10)
Survival rate N (%)	26 (92.9)
Oxygen requirement N (%)^#^	23 (95.8)^#^
COVID-19-pneumonia N (%)	23 (82.1)

N, number; SD, standard derivation; ^§^COVID-19-Severity Score (WHO) (WHO Working Group on the Clinical Characterisation and Management of COVID-19 infection, 2020) ranging from 0 (not infected) to 10 (dead); ^#^Data on oxygen requirement were only available for 24 out of the total 28 study participants.

Although all study participants had an earlier positive SARS-CoV-2 PCR, we only detected virus in nasopharyngeal samples of 22 participants after study inclusion (**Table 2**). Of these 22 patients with nasopharyngeal PCR, 5 were positive in one of the two ocular samples and one in both eye samples. We observed a tendency for lower CT-values (mean 19.6 and 19.8), i.e. more virus, in nasopharyngeal swabs of individuals with at least one PCR positive eye, compared to those patients where both eyes were negative (mean CT-value 28.4). In 6 individuals no SARS-CoV-2 was detected in any sample.

**Table 2:** SARS-CoV-2 PCR results of nasopharyngeal and conjunctival swabs.

Patients with viral load detectable in	Number (%)	Nasopharyngeal swabs Mean CT ± SD (range)	Conjunctival swabs Mean CT ± SD (range)
neither nasopharynx nor eye	6 (21.4%)	≥40	≥40
only nasopharynx	16 (57.1%)	28.4 ± 3.8 (22.1-34.3)	≥40
nasopharynx + one eye	5 (17.9%)	19.6 ± 2.8 (16.4-23.6)	35.2 ± 2.6 (30.9-37.1)
nasopharynx + both eyes	1 (3.6%)	19.8	35.9 ± 2.4 (34.2-37.6)

At the time of our study, mainly D614G and Alpha variants circulated in Austria. To determine infecting variants in study participants and compare virus in nasopharyngeal and ocular samples, we analyzed all PCR positive samples using nanopore sequencing. Virus in nasopharyngeal samples was assigned to the D614G variant for 4 samples, while 11 participants were infected with the Alpha variant. For 7 positive nasopharyngeal samples sequencing was not possible, probably due to high CT-values and consequently low vial load in the samples. Quality and thus coverage of sequences for ocular samples was generally reduced, probably due to lower viral load in these samples compared to the corresponding nasopharyngeal samples (sequences can be found as **Supplementary Material**). However, nasopharyngeal and ocular sequences from the same patient were assigned to the same variant.

**Figure 1:**
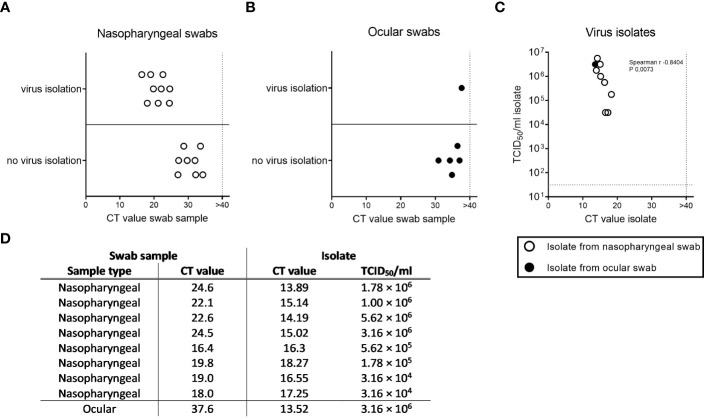
Virus recovery from nasopharyngeal and ocular swab samples. Infectious virus was attempted to recovered from PCR positive nasopharyngeal (**A**, n=17) and ocular (**B**, n=6) samples. Virus isolation was considered successful when clear cytopathic effect (CPE) was visible latest after two passages and negative when no CPE was visible after two passages. Presence of SARS-CoV-2 in culture supernatant was confirmed via PCR. Shown is success of virus recovery relative to CT-value of initial nasopharyngeal or ocular swab sample. **(C)** Successful virus isolates for 8 nasopharyngeal and 1 ocular sample were analyzed for total virus via PCR and infectious virus via TCID_50_ assay. CT-values ≥ 40 were considered negative (vertical dotted lines). Horizontal dotted line in panel **(C)** indicated limit of detection for TCID_50_ assay (31.6 TCID_50_/ml). Non-parametric correlation was determined according to Spearman. **(D)** Table summarizing characteristics of isolates shown in Panel **(C)** and underlying swab samples.

To verify whether PCR positive samples contain infectious virus, we performed co-culture experiment on Vero-TMPRSS2/ACE2 cells highly susceptible for SARS-CoV-2. We attempted to isolate virus for a total of 23 PCR positive samples with enough material remaining (17 nasopharyngeal and 6 ocular samples). We were able to successfully propagate SARS-CoV-2 from all nasopharyngeal swabs with a CT-value below 27 (n=9), while this was not the case for those with higher CT-values (**Figure 1A**). This is in line with other studies showing an increased isolation success with decreasing CT-values of swab samples (Berengua et al., 2022; Fomenko et al., 2022). Surprisingly, we also generated a SARS-CoV-2 isolate from one of the six conjunctival swabs, which to our knowledge has not been described in the literature yet (**Figure 1B**) (Ho et al., 2020; Liang and Wu, 2020; Wu et al., 2020; Zhou Y. et al., 2020). Sequence analysis confirmed that this patient was infected with an Alpha variant. To confirm successful isolation we performed PCR and TCID_50_ assays from culture supernatants for 8 of the nasopharyngeal isolates and the ocular isolate (**Figures 1C, D**). For all samples infectious virus was detected in the TCID_50_ assay and infectious viral titers negatively correlated with the CT-value (total virus). We further compared virus sequences of nasopharyngeal and conjunctival samples and derived SARS-CoV-2 isolates. Samples derived from the same patient correlated well, with the limitation of reduced sequence quality for the conjunctival samples.

## 4 Discussion

In our study, we detected SARS-CoV-2 in the eye of about 27% of patients with a positive nasopharyngeal swab sample. This rate was higher than in a recent meta-analysis that found 0-8% and 0-5.3% of tear samples positive for SARS-CoV-1 and SARS-CoV-2, respectively (Al-Sharif et al., 2021). Another study analyzing post-mortem eye samples also found lower rates of patients with positive eyes (Sawant et al., 2021). Infection of the eye may be highly dependent on factors such as infecting variant, respiratory viral load, severity of disease, symptoms and patient characteristics. We observed a tendency for lower CT-values, i.e. higher viral load, in the nasopharyngeal samples of patients with virus detectable in the eye compared to those with negative ocular swabs. Additionally, we included only hospitalized patients for which time since initial positive PCR might be longer compared to the other studies.

Conjunctivitis as a symptom associated with SARS-CoV-2 infection has been described with variable frequency (Güemes-Villahoz et al., 2020; Guan et al., 2020; Wu et al., 2020). Virus replication in the eye might be higher in patients with conjunctivitis, however little data is available on this. Virus may be introduced into the eye from the surface via respiratory droplets or hand-to-eye contact or be transported from the upper respiratory track into the eye via the nasolacrimal duct (Collin et al., 2021). Both modes of eye infection might be enhanced by the increased replication of Omicron variants in the upper respiratory track compared to earlier variants. Infection of cells with Omicron was shown to occur via the endocytic pathway rather than direct fusion on the plasma membrane, and was therefore less dependent on TMPRSS2 expression in the cells (Hui et al., 2022; Peacock et al., 2022). Consequently, also TMPRSS2 negative ACE2 expressing cells in the eye might be infected by Omicron variants. A recent study performed during the XBB.1.16 wave in India finds an increased ocular manifestation of SARS-CoV-2 infection in young infants with conjunctivitis in around one third of these children (Vashishtha and Kumar, 2023).

Infection of the eye is also known for other respiratory viruses such as influenza, RSV or adenoviruses and these infections might cause conjunctivitis (Belser et al., 2013, 2018). Here again the anatomical link of the eye and the respiratory track via the nasolacrimal duct and expression of receptors mediating virus entry in the eye may facilitate spread and replication of respiratory viruses in the ocular compartment. For influenza a broad range of virus isolates can infect primary human corneal epithelial cells *in vitro*, however, non-ocular isolates were more efficiently inhibited by tears compared to ocular isolates (Creager et al., 2018). In our study we generated one virus isolate from an ocular swab and did not find major sequence differences compared to the matching nasopharyngeal isolate. However, further studies are needed to detect potential differences in ocular tropism of different SARS-CoV-2 variants.

Our study has a number of limitations such as the small number of patients, the fact that only hospitalized patients were included and that no information regarding conjunctivitis were collected. Additionally replication of virus in the eye and transmission of the virus might be difficult to distinguish. Nevertheless, our data and other studies (Xia et al., 2020; Xie et al., 2020) indicate that also ocular fluids could be infectious and therefore may represent a potential source of virus transmission. This has especially implications for ophthalmologist practices and emphasizes on the need for appropriate protective equipment and vigorous disinfection protocols for instruments and equipment. Additionally, infection of eyes has practical consequences for cornea transplantation where exclusion of SARS-CoV-2 infection in donors prior to transplantation will remain crucial (Sawant et al., 2021). As conjunctivitis associated with SARS-CoV-2 infection has been described particularly for newer virus variants, further studies analyzing patients infected with currently circulating Omicron variants will be of interest.

## Data availability statement

The raw data supporting the conclusions of this article will be made available by the authors, without undue reservation.

## Ethics statement

The studies involving humans were approved by Ethical Committee of the Medical University of Innsbruck, Austria. The studies were conducted in accordance with the local legislation and institutional requirements. The participants provided their written informed consent to participate in this study. Ethical approval was not required for the studies on animals in accordance with the local legislation and institutional requirements because only commercially available established cell lines were used.

## Author contributions

JK: Conceptualization, Formal analysis, Investigation, Methodology, Project administration, Resources, Supervision, Visualization, Writing – original draft, Writing – review & editing. AR: Data curation, Investigation, Writing – original draft, Writing – review & editing. DB: Data curation, Formal analysis, Investigation, Writing – review & editing. WB: Formal analysis, Supervision, Writing – review & editing. DV: Funding acquisition, Resources, Supervision, Writing – review & editing. CZ: Conceptualization, Project administration, Resources, Writing – review & editing. TR: Data curation, Formal analysis, Writing – review & editing. SS: Data curation, Investigation, Project administration, Writing – review & editing. BF: Conceptualization, Data curation, Formal analysis, Investigation, Methodology, Project administration, Resources, Supervision, Visualization, Writing – original draft, Writing – review & editing.

## References

[B1] Al-Sharif E. Strianese D. AlMadhi N. H. D’Aponte A. dell’Omo R. Di Benedetto R.. (2021). Ocular tropism of coronavirus (CoVs): a comparison of the interaction between the animal-to-human transmitted coronaviruses (SARS-CoV-1, SARS-CoV-2, MERS-CoV, CoV-229E, NL63, OC43, HKU1) and the eye. Int. Ophthalmol. 41, 349–362. doi: 10.1007/s10792-020-01575-2 32880786 PMC7471531

[B2] Artic Network. Available online at: https://artic.network/ncov-2019 (Accessed 2023).

[B3] ARTIC SARS-CoV-2 workflow and reporting. Available online at: https://github.com/epi2me-labs/wf-artic (Accessed 2023).

[B4] Belser J. A. Lash R. R. Garg S. Tumpey T. M. Maines T. R. (2018). The eyes have it: influenza virus infection beyond the respiratory tract. Lancet Infect. Dis. 18, e220–e227. doi: 10.1016/s1473-3099(18)30102-6 29477464 PMC6035055

[B5] Belser J. A. Rota P. A. Tumpey T. M. (2013). Ocular tropism of respiratory viruses. Microbiol. Mol. Biol. Rev. 77, 144–156. doi: 10.1128/mmbr.00058-12 23471620 PMC3591987

[B6] Berengua C. López M. Esteban M. Marín P. Ramos P. Cuerpo M. D.. (2022). Viral culture and immunofluorescence for the detection of SARS-CoV-2 infectivity in RT-PCR positive respiratory samples. J. Clin. Virol. 152, 105167. doi: 10.1016/j.jcv.2022.105167 35523105 PMC9046102

[B7] Cavalleri M. Brambati M. Starace V. Capone L. Nadin F. Pederzolli M.. (2020). Ocular features and associated systemic findings in SARS-CoV-2 infection. Ocul. Immunol. Inflammation 28, 916–921. doi: 10.1080/09273948.2020.1781198 32870738

[B8] Collin J. Queen R. Zerti D. Dorgau B. Georgiou M. Djidrovski I.. (2021). Co-expression of SARS-CoV-2 entry genes in the superficial adult human conjunctival, limbal and corneal epithelium suggests an additional route of entry via the ocular surface. Ocul. Surf. 19, 190–200. doi: 10.1016/j.jtos.2020.05.013 32502616 PMC7267807

[B9] Creager H. M. Kumar A. Zeng H. Maines T. R. Tumpey T. M. Belser J. A. (2018). Infection and replication of influenza virus at the ocular surface. J. Virol. 92, e02192-17. doi: 10.1128/jvi.02192-17 29321303 PMC5972870

[B10] Eurosurveillance Editorial Team (2020). Note from the editors: World Health Organization declares novel coronavirus, (2019-nCoV) sixth public health emergency of international concern. Euro Surveill. 25. doi: 10.2807/1560-7917.ES.2020.25.5.200131e PMC7014669 32019636

[B11] Ewels P. A. Peltzer A. Fillinger S. Patel H. Alneberg J. Wilm A.. (2020). The nf-core framework for community-curated bioinformatics pipelines. Nat. Biotechnol. 38, 276–278. doi: 10.1038/s41587-020-0439-x 32055031

[B12] Fomenko A. Weibel S. Moezi H. Menger K. Schmucker C. Metzendorf M. I.. (2022). Assessing severe acute respiratory syndrome coronavirus 2 infectivity by reverse-transcription polymerase chain reaction: A systematic review and meta-analysis. Rev. Med. Virol. 32, e2342. doi: 10.1002/rmv.2342 35366033 PMC9111068

[B13] Freed N. E. Vlková M. Faisal M. B. Silander O. K. (2020). Rapid and inexpensive whole-genome sequencing of SARS-CoV-2 using 1200 bp tiled amplicons and Oxford Nanopore Rapid Barcoding. Biol. Methods Protoc. 5, bpaa014. doi: 10.1093/biomethods/bpaa014 33029559 PMC7454405

[B14] Guan W. J. Ni Z. Y. Hu Y. Liang W. H. Ou C. Q. He J. X.. (2020). Clinical characteristics of coronavirus disease 2019 in China. N Engl. J. Med. 382, 1708–1720. doi: 10.1056/NEJMoa2002032 32109013 PMC7092819

[B15] Güemes-Villahoz N. Burgos-Blasco B. García-Feijoó J. Sáenz-Francés F. Arriola-Villalobos P. Martinez-de-la-Casa J. M.. (2020). Conjunctivitis in COVID-19 patients: frequency and clinical presentation. Graefes Arch. Clin. Exp. Ophthalmol. 258, 2501–2507. doi: 10.1007/s00417-020-04916-0 32860573 PMC7455778

[B16] Harthaller T. Borena W. Bante D. Schäfer H. Strallhofer O. Zöggeler T.. (2022). High prevalence of undocumented SARS-CoV-2 infections in the pediatric population of the Tyrolean district of Schwaz. Viruses 14, 1–12. doi: 10.3390/v14102294 PMC9609860 36298849

[B17] Ho D. Low R. Tong L. Gupta V. Veeraraghavan A. Agrawal R. (2020). COVID-19 and the ocular surface: A review of transmission and manifestations. Ocul. Immunol. Inflammation 28, 726–734. doi: 10.1080/09273948.2020.1772313 32543262

[B18] Hui K. P. Y. Ho J. C. W. Cheung M.-C. Ng K.-C. Ching R. H. H. Lai K.-L.. (2022). SARS-CoV-2 Omicron variant replication in human bronchus and lung ex vivo. Nature 603, 715–720. doi: 10.1038/s41586-022-04479-6 35104836

[B19] Lan J. Ge J. Yu J. Shan S. Zhou H. Fan S.. (2020). Structure of the SARS-CoV-2 spike receptor-binding domain bound to the ACE2 receptor. Nature 581, 215–220. doi: 10.1038/s41586-020-2180-5 32225176

[B20] Lau S. K. Woo P. C. Yip C. C. Tse H. Tsoi H. W. Cheng V. C.. (2006). Coronavirus HKU1 and other coronavirus infections in Hong Kong. J. Clin. Microbiol. 44, 2063–2071. doi: 10.1128/jcm.02614-05 16757599 PMC1489438

[B21] Leong H. N. Chan K. P. Khan A. S. Oon L. Se-Thoe S. Y. Bai X. L.. (2004). Virus-specific RNA and antibody from convalescent-phase SARS patients discharged from hospital. Emerg. Infect. Dis. 10, 1745–1750. doi: 10.3201/eid1010.040026 15504259 PMC3323266

[B22] Liang L. Wu P. (2020). There may be virus in conjunctival secretion of patients with COVID-19. Acta Ophthalmol. 98, 223. doi: 10.1111/aos.14413 32189460 PMC7228356

[B23] Loon S. C. Teoh S. C. Oon L. L. Se-Thoe S. Y. Ling A. E. Leo Y. S.. (2004). The severe acute respiratory syndrome coronavirus in tears. Br. J. Ophthalmol. 88, 861–863. doi: 10.1136/bjo.2003.035931 15205225 PMC1772213

[B24] Peacock T. P. Brown J. C. Zhou J. Thakur N. Sukhova K. Newman J.. (2022). The altered entry pathway and antigenic distance of the SARS-CoV-2 Omicron variant map to separate domains of spike protein. bioRxiv 2021, 2012.2031.474653. doi: 10.1101/2021.12.31.474653

[B25] Riepler L. Rössler A. Falch A. Volland A. Borena W. von Laer D.. (2020). Comparison of four SARS-CoV-2 neutralization assays. Vaccines (Basel) 9, 1–14. doi: 10.3390/vaccines9010013 33379160 PMC7824240

[B26] Sawant O. B. Singh S. Wright R. E. 3rd Jones K. M. Titus M. S. Dennis E.. (2021). Prevalence of SARS-CoV-2 in human post-mortem ocular tissues. Ocul. Surf. 19, 322–329. doi: 10.1016/j.jtos.2020.11.002 33176215 PMC7649030

[B27] Senanayake P. Drazba J. Shadrach K. Milsted A. Rungger-Brandle E. Nishiyama K.. (2007). Angiotensin II and its receptor subtypes in the human retina. Invest. Ophthalmol. Vis. Sci. 48, 3301–3311. doi: 10.1167/iovs.06-1024 17591902

[B28] van der Hoek L. Pyrc K. Jebbink M. F. Vermeulen-Oost W. Berkhout R. J. Wolthers K. C.. (2004). Identification of a new human coronavirus. Nat. Med. 10, 368–373. doi: 10.1038/nm1024 15034574 PMC7095789

[B29] Vashishtha V. M. Kumar P. (2023). Conjunctival involvement in infants as an unusual symptom of omicron XBB.1.16 driven surge. Indian Pediatr. 60, 861–862. doi: 10.1007/s13312-023-3020-0 37818812

[B30] WHO Working Group on the Clinical Characterisation and Management of COVID-19 infection (2020). A minimal common outcome measure set for COVID-19 clinical research. Lancet Infect. Dis. 20, e192–e197. doi: 10.1016/s1473-3099(20)30483-7 32539990 PMC7292605

[B31] Wu P. Duan F. Luo C. Liu Q. Qu X. Liang L.. (2020). Characteristics of ocular findings of patients with coronavirus disease 2019 (COVID-19) in Hubei Province, China. JAMA Ophthalmol. 138, 575–578. doi: 10.1001/jamaophthalmol.2020.1291 32232433 PMC7110919

[B32] Xia J. Tong J. Liu M. Shen Y. Guo D. (2020). Evaluation of coronavirus in tears and conjunctival secretions of patients with SARS-CoV-2 infection. J. Med. Virol. 92, 589–594. doi: 10.1002/jmv.25725 32100876 PMC7228294

[B33] Xie H. T. Jiang S. Y. Xu K. K. Liu X. Xu B. Wang L.. (2020). SARS-CoV-2 in the ocular surface of COVID-19 patients. Eye Vis. (Lond.) 7, 23. doi: 10.1186/s40662-020-00189-0 32355863 PMC7183451

[B34] Zaki A. M. van Boheemen S. Bestebroer T. M. Osterhaus A. D. Fouchier R. A. (2012). Isolation of a novel coronavirus from a man with pneumonia in Saudi Arabia. N Engl. J. Med. 367, 1814–1820. doi: 10.1056/NEJMoa1211721 23075143

[B35] Zhong N. S. Zheng B. J. Li Y. M. Poon Xie Z. H. Chan K. H.. (2003). Epidemiology and cause of severe acute respiratory syndrome (SARS) in Guangdong, People’s Republic of China, in February 2003. Lancet 362, 1353–1358. doi: 10.1016/s0140-6736(03)14630-2 14585636 PMC7112415

[B36] Zhou Y. Duan C. Zeng Y. Tong Y. Nie Y. Yang Y.. (2020). Ocular findings and proportion with conjunctival SARS-COV-2 in COVID-19 patients. Ophthalmology 127, 982–983. doi: 10.1016/j.ophtha.2020.04.028 32359840 PMC7194804

[B37] Zhou P. Yang X. L. Wang X. G. Hu B. Zhang L. Zhang W.. (2020). A pneumonia outbreak associated with a new coronavirus of probable bat origin. Nature 579, 270–273. doi: 10.1038/s41586-020-2012-7 32015507 PMC7095418

